# Supplementary material for: The Growing Altitude Influences the Flavor Precursors, Sensory Characteristics and Cupping Quality of the Pu’er Coffee Bean
Source: Foods. 2024 Nov 28;13(23):3842. doi: 10.3390/foods13233842 (PMC11640416; doi:10.3390/foods13233842)

Supplementary material - Table S1

NO.	Chemical compounds	Retention time (min)	CAS	Content (μg/g)				
				A	B	C	D	E
	Alcohols							
1	3-pentanol	19.67	584-02-1	66.07±5.41	69.48±2.46	66.91±3.40	64.19±1.37	63.82±3.41
2	1-hexanol	21.19	111-27-3	5.42±0.26	4.39±0.01	3.73±0.78	3.95±0.58	3.92±0.32
3	(Z)-linalool oxide (furanoid)	27.63	5989-33-3	8.17±1.46	7.52±0.71	9.13±0.59	8.42±0.33	8.22±0.36
4	Linalool	32.62	78-70-6	5.47±0.89	4.33±0.10	7.71±1.38	6.78±0.17	6.23±0.62
5	2-furanmethanol	38.66	98-00-0	2636.28±35.58	1115.85±39.96	914.43±21.75	251.89±10.32	247.49±44.46
6	2-furanmethanol, 5-methyl-	41.99	3857-25-8	11.71±0.81	10.09±0.47	10.37±1.13	7.97±0.66	8.17±0.47
7	Benzeneethanol	51.25	60-12-8	11.50±0.22	10.12±0.18	5.93±0.76	9.34±0.50	9.11±0.34
8	2-thiophenemethanol	52.85	636-72-6	5.06±1.59	11.78±0.42	12.64±1.12	11.59±0.9	3.86±0.33
	Aldehydes							
1	Furfural	26.83	98-01-1	3928.86±81.58	4435.52±32.59	4444.71±47.19	4700.98±40.79	4863.42±13.14
2	Benzaldehyde	29.76	100-52-7	24.48±2.79	21.88±0.71	20.39±1.23	21.04±0.43	17.10±1.00
3	2-furancarboxaldehyde, 5-methyl-	33.03	620-02-0	2974.00±68.91	3136.43±36.34	3006.95±164.12	3261.21±50.69	3208.80±112.25
4	Benzeneacetaldehyde	36.55	122-78-1	54.50±3.64	57.82±1.24	75.24±0.60	77.91±1.62	71.96±1.11
5	2-thiophenecarboxaldehyde	39.35	98-03-3	28.16±2.05	26.09±0.49	27.75±1.15	32.39±4.36	24.32±3.69
6	2-phenyl-2-butenal	51.76	4411-89-6	2.66±0.37	2.32±0.16	2.23±0.44	2.32±0.44	2.36±0.48
7	1H-pyrrole-2-carboxaldehyde	56.40	1003-29-8	142.72±12.22	134.42±2.21	151.94±1.47	177.55±3.16	154.55±2.89

	Acids							
1	Acetic acid	26.00	64-19-7	1805.09±52.55	1951.06±23.73	1801.41±26.72	1778.92±12.83	1997.77±27.16
2	3-methylbutanoic acid	39.05	503-74-2	384.01±6.54	514.65±4.44	384.96±4.29	521.55±5.26	455.82±2.07
3	2-butenoic acid, 3-methyl-	45.62	541-47-9	55.71±3.69	85.75±0.80	66.66±1.03	83.24±1.31	86.82±1.58
	Esters							
1	Propanoic acid, ethenyl ester	31.07	105-38-4	508.15±10.90	497.99±5.38	507.10±9.73	512.51±5.70	497.37±6.39
2	2-furanmethanol, acetate	31.51	623-17-6	406.92±8.09	403.78±3.18	370.99±4.13	373.77±5.23	357.87±3.58
3	2-furanmethanol, propanoate	35.00	623-19-8	8.82±0.61	8.41±0.35	7.34±0.33	7.07±0.14	6.60±0.11
4	γ-butyrolactone	35.40	96-48-0	109.04±24.01	98.29±2.48	99.83±6.28	110.33±3.59	91.88±4.49
	Ketones							
1	2,3-butanedione	4.58	431-03-8	290.86±48.27	341.12±9.44	275.56±32.07	70.13±4.27	305.84±13.13
2	2,3-pentanedione	6.68	600-14-6	457.72±49.79	539.97±26.90	375.80±117.50	388.03±121.55	460.62±90.87
3	3-penten-2-one	8.87	625-33-2	23.67±0.99	19.03±2.92	21.53±0.90	18.61±1.52	17.95±1.30
4	2,3-hexanedione	9.24	3848-24-6	35.12±2.23	30.45±2.62	31.76±4.48	30.27±3.49	31.38±3.28
5	3,4-hexanedione	9.64	4437-51-8	49.87±3.54	49.87±3.15	45.64±2.29	44.36±0.74	43.39±4.12
6	2-methyloxolan-3-one	15.42	3188-00-9	215.94±12.89	190.18±26.22	341.01±62.64	243.61±71.92	400.04±17.08
7	2-butanol-3-one	16.48	513-86-0	125.00±10.82	132.01±18.76	86.11±22.67	140.20±2.90	133.22±4.46
8	2-propanone, 1-hydroxy-	17.21	116-09-6	1898.85±53.40	1995.71±21.60	1942.64±25.04	1784.09±9.03	1974.08±31.67
9	3-hexanone, 2-methyl-	20.45	7379-12-6	27.93±1.56	27.85±1.10	26.69±1.46	21.57±2.23	20.71±1.52
10	2-hydroxy-3-pentanone	20.71	5704-20-1	65.24±1.69	65.94±2.09	63.44±2.74	61.89±1.23	58.37±3.14
11	2-methylcyclopentenone	20.96	1120-73-6	12.74±0.19	11.80±0.64	10.85±0.89	10.37±0.51	9.71±0.65

12	1-hydroxy-2-butanone	21.46	5077-67-8	198.6±3.56	224.80±0.77	225.36±3.74	211.21±1.30	204.15±26.83
13	2(3H)-furanone, 5-methyl-	24.69	591-12-8	9.94±0.16	11.16±0.46	10.78±0.54	10.48±0.49	11.50±0.30
14	2-propanone, 1-(acetyloxy)-	27.36	592-20-1	1651.10±22.78	1670.45±17.6	1734.74±39.97	1816.31±28.07	1680.65±30.65
15	2,5-hexanedione	28.99	110-13-4	27.94±1.12	30.31±0.76	29.97±2.28	28.03±0.59	27.29±3.14
16	Ethanone, 1-(2-furanyl)-	29.09	1192-62-7	976.21±23.11	1090.07±11.97	1009.22±15.23	1031.24±23.14	1089.78±76.04
17	2-cyclopenten-1-one, 3-methyl-	29.37	2758-18-1	9.99±1.00	8.56±0.86	9.60±0.88	7.72±2.05	6.35±1.57
18	3(2H)-thiophenone, dihydro-2-methyl-	30.16	13679-85-1	27.10±0.86	25.39±1.28	23.55±1.74	21.29±1.63	19.19±1.02
19	2-cyclopenten-1-one, 2,3-dimethyl-	30.73	1121-05-7	16.46±0.95	14.60±1.43	13.46±0.57	13.12±0.97	12.42±0.06
20	2-butanone, 1-(acetyloxy)-	31.31	1575-57-1	303.89±7.43	298.17±2.99	303.11±5.99	306.65±4.37	296.48±4.15
21	1-propanone, 1-(2-furanyl)-	33.21	3194-15-8	135.47±5.69	143.46±2.09	129.01±2.12	138.16±2.82	149.93±0.41
22	3,6-heptanedione	33.32	1703-51-1	30.32±1.54	30.86±3.25	30.47±2.12	27.21±0.20	24.87±7.91
23	4-cyclopentene-1,3-dione	33.38	930-60-9	48.78±4.57	63.39±1.99	65.51±2.24	61.99±4.71	77.39±0.93
24	Ethanone, 1-(2-pyridinyl)-	34.44	1122-62-9	18.81±2.13	14.58±0.33	18.65±7.8	17.65±5.46	16.68±6.29
25	Ethanone, 1-(1-methyl-1H-pyrrol-2-yl)-	37.48	932-16-1	67.85±3.45	59.14±0.94	60.74±0.93	53.16±7.84	43.93±0.78
26	3-penten-2-one, 3-methyl-	38.35	565-62-8	11.01±5.42	8.55±0.13	8.85±2.38	8.40±0.74	11.52±4.83
27	Ethylcyclopentenolone	38.92	21835-01-8	52.38±2.50	55.69±0.77	48.84±1.18	50.34±1.32	51.71±0.63
28	1-(5-methyl-2-pyrazinyl)-1-ethanone	38.94	22047-27-4	46.98±3.73	34.98±2.56	37.51±0.67	31.83±6.30	31.56±0.82
29	1-(6-methyl-2-pyrazinyl)-1-ethanone	39.39	22047-26-3	7.66±1.44	6.60±0.14	81.53±0.88	12.59±1.08	65.93±1.10
30	2,5-furandione, 3,4-dimethyl-	41.14	766-39-2	32.67±2.21	34.09±3.71	28.78±3.79	29.89±2.82	32.28±0.92

31	2(5H)-furanone	42.28	497-23-4	101.07±5.35	106.13±0.60	101.60±1.90	104.83±1.06	104.69±1.57
32	Ethanone, 1-(2-thienyl)-	43.72	88-15-3	16.66±2.51	13.33±0.36	13.43±1.18	14.31±0.27	10.23±1.09
33	1,2-cyclopentanedione	43.85	3008-40-0	18.57±0.70	19.39±0.62	22.19±1.18	21.50±0.29	18.40±0.44
34	2H-pyran-2-one, 6-ethyltetrahydro-	44.66	3301-90-4	7.63±1.88	6.17±0.51	5.44±0.17	6.98±0.16	6.30±0.38
35	2-cyclopenten-1-one, 2-hydroxy-3,4-dimethyl-	45.47	21835-00-7	19.38±1.69	18.17±1.01	16.93±0.76	17.80±0.42	17.98±0.25
36	Methyl cyclopentenolone	47.01	80-71-7	109.85±6.98	126.79±1.30	125.25±1.01	132.24±1.91	136.11±2.52
37	4-hydroxy-2,5-dimethylfuran-3-one	48.38	3658-77-3	109.17±6.46	125.19±3.45	130.72±2.05	105.82±1.08	150.17±4.07
38	Ethanone, 1-(1H-pyrrol-2-yl)-	54.06	1072-83-9	141.40±3.73	111.48±2.49	113.15±7.88	132.77±11.86	126.25±3.97
39	5-acetyloxolan-2-one	57.85	29393-32-6	34.52±4.27	35.23±1.76	35.62±3.30	35.65±0.82	40.39±1.03
40	4H-pyran-4-one, 2,3-dihydro-3,5-dihydroxy-6-methyl-	67.56	28564-83-2	23.72±3.01	27.54±2.15	33.34±3.43	17.93±2.91	36.52±4.60
	Ethers							
1	4-Methylanisole	16.98	104-93-8	28.88±5.40	32.33±1.48	26.19±1.52	26.34±3.94	33.27±2.66
	Thiophenes							
1	3-acetyl-2,5-dimethylthiophene	45.83	2530-10-1	13.30±0.24	11.50±0.35	13.62±0.45	10.50±0.56	10.06±0.71
	Pyrazines							
1	Pyrazine	12.69	290-37-9	154.13±61.57	160.82±4.77	238.96±8.84	168.13±69.83	196.88±6.2
2	Pyrazine, methyl-	15.53	109-08-0	3237.73±26.78	2706.26±40.63	2613.03±79.7	168.53±57.97	146.15±52.99
3	Pyrazine, 2,5-dimethyl-	18.68	123-32-0	1835.68±16.88	1507.47±28.14	1644.90±31.21	1536.42±17.19	1473.48±24.34
4	Pyrazine, 2,6-dimethyl-	19.06	108-50-9	482.22±89.72	301.40±10.03	307.20±21.41	74.73±0.59	67.57±3.38

5	Pyrazine, ethyl-	19.35	13925-00-3	742.84±7.78	600.25±11.33	676.47±11.96	631.55±7.50	537.83±12.63
6	Pyrazine, 2,3-dimethyl-	19.99	5910-89-4	294.10±3.19	223.38±4.84	237.37±5.61	237.65±2.41	199.79±5.70
7	Pyrazine, 2-ethyl-6-methyl-	22.40	13925-03-6	1100.64±14.22	846.00±10.29	907.12±5.65	841.35±7.68	757.84±5.76
8	Pyrazine, 2-ethyl-5-methyl-	22.71	13360-64-0	376.90±6.89	291.23±4.06	556.35±3.26	473.46±64.8	272.79±3.30
9	Pyrazine, 2-ethyl-3-methyl-	23.48	15707-23-0	791.55±16.53	583.90±8.8	622.92±3.14	601.66±6.69	548.74±2.46
10	Pyrazine, 2-(n-propyl)-	24.22	18138-03-9	29.95±1.79	24.89±2.02	25.35±1.63	23.23±0.58	15.77±1.74
11	Pyrazine, ethenyl-	25.19	4177-16-6	65.00±4.18	53.90±1.36	56.35±3.66	56.89±1.50	47.83±1.23
12	Pyrazine, 2,6-diethyl-	25.31	13067-27-1	108.76±3.28	74.55±1.04	80.77±0.76	70.06±4.39	61.13±0.32
13	Pyrazine, 3-ethyl-2,5-dimethyl-	26.08	13360-65-1	543.57±16.77	364.93±6.8	383.13±5.35	355.42±7.88	334.49±5.80
14	2,3-diethylpyrazine	26.50	15707-24-1	8.56±0.42	5.72±0.17	6.08±0.21	5.20±0.26	4.07±0.71
15	Pyrazine, 2-methyl-6-propyl-	27.09	29444-46-0	27.11±1.13	17.67±2.75	20.10±0.26	20.15±0.62	16.71±0.43
16	Pyrazine, 1-methylethenyl-	28.08	38713-41-6	4.24±0.21	2.88±0.04	3.33±0.28	3.16±0.12	2.28±0.05
17	Pyrazine, 2-ethenyl-6-methyl-	28.26	13925-09-2	102.68±5.65	81.18±2.07	86.85±1.50	81.85±1.99	77.05±0.63
18	Pyrazine, 2-ethenyl-5-methyl-	28.61	13925-08-1	62.63±3.25	53.43±7.25	52.49±1.83	48.35±1.18	47.17±0.71
19	Pyrazine, 3,5-diethyl-2-methyl-	28.87	18138-05-1	75.30±3.48	46.44±1.05	49.99±0.50	40.38±7.53	37.12±4.79
20	Pyrazine, (1-methylethenyl)-	34.23	38713-41-6	36.84±2.91	26.89±0.88	30.70±0.42	32.40±1.94	26.95±4.54
21	Acetylpyrazine	35.80	22047-25-2	47.73±3.08	35.30±1.22	41.00±1.28	39.75±0.65	31.37±0.62
22	Pyrazine, 2-methyl-6-(1-propenyl)-, (Z)-	37.55	55138-67-5	13.14±0.94	8.33±0.23	10.07±0.15	9.16±0.49	7.53±0.21
23	Pyrazine, 2-methyl-5-(1-propenyl)-, (E)-	40.65	18217-82-8	25.77±2.25	15.67±0.77	16.04±3.16	18.90±1.05	15.83±0.45
24	4-methylpyrrolo[1,2-a]pyrazine	55.01	64608-60-2	8.06±1.20	5.72±0.66	2.98±0.32	6.20±0.75	2.92±0.24

	Pyrroles							
1	1-methylpyrrole	9.42	96-54-8	39.96±4.18	25.39±2.55	29.86±1.31	27.88±0.62	20.11±1.42
2	Pyrrole	29.95	109-97-7	68.26±1.24	45.69±1.45	48.09±2.61	49.93±4.57	31.47±1.12
3	1H-pyrrole-2-carboxaldehyde, 1-methyl-	35.37	1192-58-1	177.93±6.49	168.4±2.35	180.76±2.52	195.08±3.66	153.54±1.64
4	1H-pyrrole, 1-(2-furanylmethyl)-	46.99	1438-94-4	76.81±5.14	68.99±5.75	67.99±0.60	70.83±1.65	60.92±1.68
	Pyridines							
1	Pyridine	11.31	110-86-1	1042.89±20.83	850.40±8.69	809.92±5.61	478.12±34.82	374.91±26.68
2	Pyridine, 3-ethyl-	21.94	536-78-7	17.60±3.81	13.00±1.96	12.34±2.97	16.80±0.62	9.39±0.54
	Phenols							
1	Phenol, 2-methoxy-	48.56	90-05-1	40.10±2.84	33.69±0.62	36.04±0.53	42.59±0.73	31.22±1.03
2	Maltol	53.56	118-71-8	246.62±30.13	238.34±11.87	254.7±21.73	291.92±12.91	265.04±17.37
3	Phenol	55.93	108-95-2	25.75±2.56	21.18±4.18	27.20±1.09	27.79±0.50	19.24±2.95
4	Phenol, 2-methyl-	56.02	95-48-7	4.52±0.43	4.59±0.07	4.39±0.19	4.46±0.16	4.28±0.18
5	Phenol, 4-ethyl-2-methoxy-	56.97	2785-89-9	15.94±1.35	10.68±0.18	12.67±0.35	13.89±0.98	9.36±0.31
6	Phenol, 4-methyl-	59.92	106-44-5	7.75±0.61	7.37±0.11	6.38±0.18	7.15±0.82	8.22±0.50
7	2-methoxy-4-vinylphenol	64.47	7786-61-0	127.63±13.18	111.19±5.97	136.52±10.96	130.8±7.37	112.87±10.57
	Furans							
1	2-vinylfuran	7.14	1487-18-9	16.98±2.67	7.01±0.76	36.14±3.19	7.26±1.42	6.75±0.95
2	Furan, 2-(methoxymethyl)-	14.28	13679-46-4	10.25±2.66	30.42±0.77	22.75±3.36	22.51±6.12	26.41±2.01

3	Furan, 2-[(methylthio)methyl]-	28.16	1438-91-1	15.22±0.59	15.15±0.32	12.71±1.00	16.87±0.41	13.02±0.24
4	Furan, 2-butyl-	30.19	4466-24-4	93.10±2.80	100.84±0.68	90.96±2.88	99.20±1.56	98.91±1.91
5	Benzofuran, 2-methyl-	33.95	4265-25-2	5.63±0.55	5.73±0.18	5.02±0.14	4.90±0.17	5.34±0.27
6	2,2'-bifuran	34.36	5905-00-0	39.11±2.19	41.21±0.91	33.80±2.25	31.85±0.71	40.54±0.71
7	2-acetyl-5-methylfuran	35.28	1193-79-9	19.19±1.39	18.50±0.33	17.06±0.62	17.67±0.40	17.11±0.28
8	Furan, 2,2'-methylenebis-	35.45	1197-40-6	28.34±1.69	28.85±0.40	25.72±4.31	25.55±0.45	22.49±2.40
9	2-methyl-5-propionylfuran	40.79	10599-69-6	1.10±0.14	1.25±0.14	0.91±0.04	0.88±0.05	1.06±0.20
10	Furan, 2,2'-[oxybis(methylene)]bis-	54.94	4437-22-3	16.29±1.55	14.66±0.28	13.45±0.47	14.35±0.81	12.80±0.82
11	Benzofuran, 2,3-dihydro-	72.12	496-16-2	4.27±0.26	3.98±0.33	4.16±0.31	4.13±0.22	3.65±0.33

Supplementary material - **Figure S1**

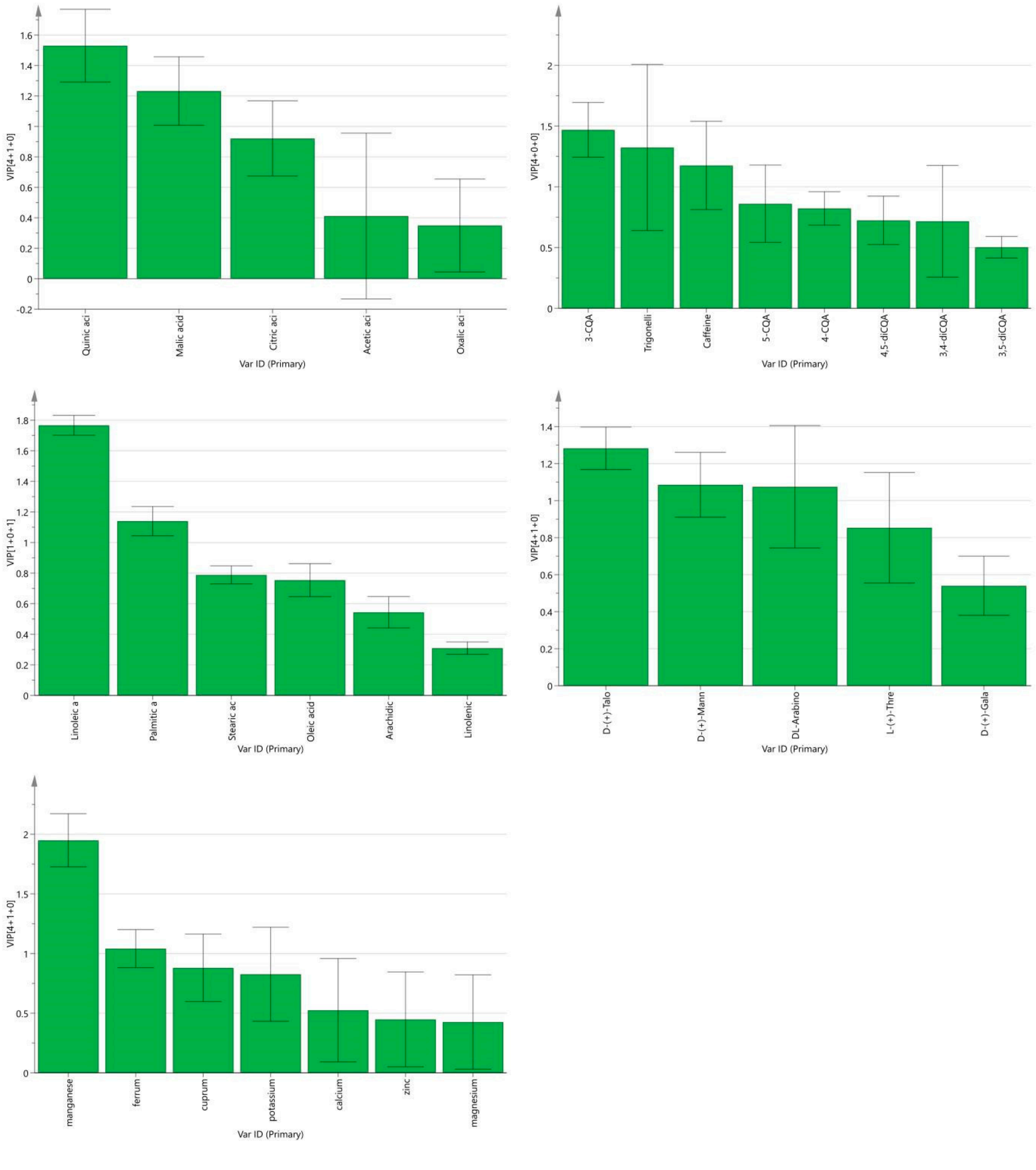

Supplement: Supplementary file 1 [file foods-13-03842-s001.zip › foods-3256406-supplementary.pdf]
